# *In utero* therapy for congenital disorders using amniotic fluid stem cells

**DOI:** 10.3389/fphar.2014.00270

**Published:** 2014-12-19

**Authors:** Durrgah L. Ramachandra, Steven S. W. Shaw, Panicos Shangaris, Stavros Loukogeorgakis, Pascale V. Guillot, Paolo De Coppi, Anna L. David

**Affiliations:** ^1^Stem Cells and Regenerative Medicine, Institute of Child Health, University College London, London, UK; ^2^Department of Obstetrics and Gynaecology, Chang Gung Memorial Hospital at Linkou, Taoyuan, Taiwan; ^3^Department of Obstetrics and Gynaecology, College of Medicine, Chang Gung University, Taoyuan, Taiwan; ^4^Prenatal Therapy, Institute for Women’s Health, University College London, London, UK; ^5^Cellular Reprogramming and Perinatal Therapy, Institute for Women’s Health, University College London, London, UK

**Keywords:** congenital disease, *in utero* therapy, stem cells, gene therapy, amniotic fluid

## Abstract

Congenital diseases are responsible for over a third of all pediatric hospital admissions. Advances in prenatal screening and molecular diagnosis have allowed the detection of many life-threatening genetic diseases early in gestation. *In utero* transplantation (IUT) with stem cells could cure affected fetuses but so far in humans, successful IUT using allogeneic hematopoietic stem cells (HSCs), has been limited to fetuses with severe immunologic defects and more recently IUT with allogeneic mesenchymal stem cell transplantation, has improved phenotype in osteogenesis imperfecta. The options of preemptive treatment of congenital diseases *in utero* by stem cell or gene therapy changes the perspective of congenital diseases since it may avoid the need for postnatal treatment and reduce future costs. Amniotic fluid stem (AFS) cells have been isolated and characterized in human, mice, rodents, rabbit, and sheep and are a potential source of cells for therapeutic applications in disorders for treatment prenatally or postnatally. Gene transfer to the cells with long-term transgenic protein expression is feasible. Recently, pre-clinical autologous transplantation of transduced cells has been achieved in fetal sheep using minimally invasive ultrasound guided injection techniques. Clinically relevant levels of transgenic protein were expressed in the blood of transplanted lambs for at least 6 months. The cells have also demonstrated the potential of repair in a range of pre-clinical disease models such as neurological disorders, tracheal repair, bladder injury, and diaphragmatic hernia repair in neonates or adults. These results have been encouraging, and bring personalized tissue engineering for prenatal treatment of genetic disorders closer to the clinic.

## INTRODUCTION

Congenital diseases attributed to about 510,000 deaths globally in 2010 (Lozano et al., 2012), and are estimated to contribute to over a third of pediatric admissions to the hospital and up to 50% of the total costs of pediatric hospital treatment (McCandless et al., 2004). Prenatal diagnosis of many congenital diseases are performed using traditional invasive techniques such as amniocentesis or chorionic villus sampling (CVS), but increasingly non-invasive methods using circulating fetal DNA in the maternal blood are feasible and available for prenatal diagnosis early in gestation (Danzer et al., 2012; Danzer and Johnson, 2014). The current options for most parents facing congenital diseases following prenatal diagnosis are either to terminate or continue with a known affected pregnancy.

Progress over the last two decades have resulted in fetal therapy being available for a small number of congenital structural anomalies such as spina bifida, identical twin placental complications, and congenital diaphragmatic hernia, using open surgical or fetoscopic interventions (Pearson and Flake, 2013). These options are currently restricted to the treatment of fetal pathophysiology and are usually performed in the second half of gestation, when pathology is already evident. There are almost no therapeutic options however for life-threatening genetic disorders which have pathology beginning *in utero*. Success with *in utero* transplantation (IUT) using allogeneic hematopoietic stem cells (HSCs), has been limited to fetuses with severe immunologic defects where there is an effective lack of immune response to allogeneic cells, and transplanted genetically normal cells have a proliferative advantage (Tiblad and Westgren, 2008). Mesenchymal stem cells (MSCs) appear to be less immunogenic than their hematopoietic counterparts (O’Donoghue and Fisk, 2004) and have shown to reduce fracture rate in a mouse model (Guillot et al., 2008) and engraft in human fetuses with osteogenesis imperfecta in an allogeneic setting (Horwitz et al., 2002). Attempts to treat diseases such as sickle cell disease (Westgren et al., 1996) with *in utero* HSC transplantation, have been unsuccessful, even where a suitably matched donor has been available. Mouse studies suggest that the immune barrier to allogeneic *in utero* HSC transplantation may be stronger than previously thought (Peranteau et al., 2007). Transplantation of autologous progenitor cells, which have been corrected for the disease, could avoid the fetal immune barrier and may prove more successful than allogenic progenitors.

Autologous progenitors can be obtained from the fetus itself. Both proliferative and differentiation potentials of amniotic fluid stem (AFS) cells has been demonstrated *in vitro* and *in vivo* (De Coppi et al., 2007; Ditadi et al., 2009). Studies exploring the potential of this stem cell source for the use in autologous or allogenic prenatal therapy of congenital diseases have been conducted in large animal models (Shaw et al., 2014). In this review, we explore the latest developments in the field of *in utero* therapy for congenital disorders such as stem cell transplantation and gene transfer using AFS and their potential clinical applications.

## AMNIOTIC FLUID AS A FETAL CELL SOURCE FOR IN UTERO THERAPY

Amniotic fluid (AF) consists of cells of fetal origin such as the amnion, skin, and respiratory system (Prusa and Hengstschläger, 2002; Tsai et al., 2004) and it can be obtained by routine clinical amniocentesis during pregnancy, a minimally invasive procedure used for prenatal diagnosis that usually takes place from 15 weeks of gestation (Gosden, 1983; Prusa and Hengstschläger, 2002; Delo et al., 2006). AF can also be collected during therapeutic amniodrainage procedures or even at cesarean section surgeries. Other fetal stem cell sources include the placenta, which can be accessed via ultrasound-guided CVS from 11 weeks of gestation or after birth yields epithelial, hematopoietic, and MSC types (Pipino et al., 2013; Jones et al., 2014). Fetal blood and the HSCs therein can also be collected from the umbilical cord in the first trimester of pregnancy by thin-gauge embryo fetoscopic-directed or ultrasound-guided blood sampling, although the long-term outcome following this procedure is not known (Chan et al., 2008).

In recent years, AFS cells have been explored in many clinical applications such as tissue engineering, cell transplantation, and gene therapy (Kaviani et al., 2001, 2003; Fauza, 2004; Tsai et al., 2004). C-Kit^+^ cells can be successfully isolated from AF, expanded with good population doublings and possess a very well characterized phenotype (Tsai et al., 2004; Delo et al., 2006; De Coppi et al., 2007; Ghionzoli et al., 2010). The surface antigen c-Kit (CD117) is known to be the receptor of stem cell factor and plays an essential role in gametogenesis, melanogenesis, and hematopoiesis (Fleischman, 1993; De Coppi et al., 2007). The successful expansion of AFS c-Kit^+^ cells has led to the finding of unique cell types such as mesenchymal (AFMSCs) and hematopoietic progenitors (Prusa and Hengstschläger, 2002; Delo et al., 2006; De Coppi et al., 2007; Ditadi et al., 2009).

Human AFS cells give rise to a variety of cell types such as osteogenic, myogenic, adipogenic, endothelial, hepatic, and neuronal origin; differentiation which has been be validated by the expression of mRNAs in lineage specific genes (Tsai et al., 2004; De Coppi et al., 2007). Rodent and murine AFS cells, like human AFS cells share similar growth properties and differentiation potential *in vitro* as well as the expression of embryonic and adult stem cell markers, respectively (De Coppi et al., 2007). AFS cells derived from human, mice, and sheep can be easily transduced without losing their characteristics (De Coppi et al., 2007; Ditadi et al., 2009; Mehta et al., 2011; Shaw and Bollini, 2011), and they possess privileged immunological characteristics that make them an ideal and reliable source for therapeutic transplantation (Ditadi et al., 2009).

The immune modulatory properties of AFS have shown resistance to natural killer (NK) cytotoxicity by inflammatory priming of AFS with interferon gamma (IFN-γ) and tumor necrosis factor alpha (TNF-α) and its ability to modulate lymphocyte proliferation according to its gestational age (Di Trapani et al., 2014). For instance, IFN-γ increases both MHC Class I and MHC Class II expression, indicating that like MSCs, they may not strongly contribute to rejection responses in allogeneic hosts (Moorefield et al., 2011). Moreover, AFS have shown to release high levels of cytokines including IL-6, MCP-2, MIP-3α, and MIP-1α when activated suggesting that they possess alternative molecular mechanisms to modulate immune response and regulation (Perin et al., 2010; Moorefield et al., 2011).

### GROWTH AND CHARACTERIZATION OF AMNIOTIC FLUID-DERIVED STEM CELLS

AFS cells have an estimated doubling time of 36 h and are grown without feeder layers (De Coppi et al., 2007). MSC subpopulations of AFS cells (AFMSCs), like other MSCs, maintain their spindle-shaped fibroblast-like morphology, their proliferation rate as well as their differentiation potential. Growth kinetics assays have shown that AFMSCs have a higher proliferation rate with an average doubling time of 25–38 h compared to bone marrow (BM) derived-MSCs that have an average doubling time of 30–90 h (Kaviani et al., 2001; Roubelakis et al., 2007). In addition, AFMSCs have a greater clonogenic potential compared to BM-MSCs (86 ± 4.3 versus 70 ± 5.1 colonies; Nadri and Soleimani, 2007). Despite the high proliferative rate of AFMSCs, they are still able to retain a normal karyotype with no evidence of tumorigenicity (Roubelakis et al., 2007). Human AFS cells express both markers of mesenchymal and pluripotent stem cells origin, such as stage-specific embryonic antigen (SSEA)-4 and Oct-4 (De Coppi et al., 2007). Once cultured in adherence however, they do not express markers of hematopoietic lineage such as CD45, CD34, and CD133 and express CD29, CD44, CD73, CD90, and CD105 (De Coppi, 2013). Interestingly, their plasticity, which is superior to adult stem cells, allow reprogramming into AFS derived induced pluripotent stem cells (iPS) with the change of the culture conditions they are exposed to (Lu et al., 2012; Moschidou et al., 2012, 2013; Pipino et al., 2014). This is particularly relevant as AFS cells can be utilized for cell banking of patient-specific pluripotent cells for potential applications in allogeneic cellular replacement therapies, pharmaceutical screening, and disease modeling (Moschidou et al., 2012, 2013).

In addition AFS cells, similarly to other fetal cells may represent the ideal source for therapy because, similarly to ES cells, they are easy to expand, and, in common with the adult counterparts, they are less controversial, not tumorigenic, readily cryopreserved for cell banking and their use can be accomplished on an autologous setting (De Coppi, 2013). The latter is particularly important in neonatal surgery, in the context of congenital malformations.

### GENE TRANSFER TO AMNIOTIC FLUID STEM CELLS

To be a successful autologous therapeutic resource for correcting genetic disease, AFS cells must be easily transduced to give high levels of therapeutic transgenic protein expression. The transduction of human AFS cells with vectors was explored with recombinant adenovirus vectors containing reporter genes such as AdHCMVsp1LacZ and AdCMV.eGFP (Grisafi et al., 2008). Human AFS cells presented a transduction efficiency of 100% when infected with 50 pfu/cell. Transduced human AFS cells maintained stemness features such as the expression of stem cell markers (SSEA4 and OCT4), adhesion and stromal molecules (CD29 and CD73) as well as adipogenic and osteogenic differentiation potential after infection (Grisafi et al., 2008). However, a decrease in SSEA4 expression, no expression of lipoprotein lipase (Lpl), an important adipogenic gene during differentiation and the expression of transcription factors Cbfa1 and PPaRγ detected only during early stages of differentiation suggests a slowdown in the differentiative progression and pluripotency after transduction.

Recently, we have shown that sheep AFS cells have the ability to be transduced using a lentivirus vector encoding the HIV-1 central polypurine tract element, the spleen focus forming virus LTR promoter, and the marker gene eGFP (63.2% efficiency). They have the ability to maintain the expression of MSC markers (CD44, CD58, and CD166) but were negative for hematopoietic, and endothelial markers (CD14, CD31, and CD45) as well as differentiate into adipogenic and osteogenic lineages (Shaw and Bollini, 2011; Shaw et al., 2014).

## IN UTERO TRANSPLANTATION

IUT involves the transplantation of cells to the fetus *in utero* (Muench, 2005) with the aim of treating congenital disorders by providing the correct stem cells (IUSCT) or gene corrected stem cells (IUSCGT). The benefits of an *in utero* approach to correcting genetic disease includes the prevention of pathology when it arises antenatally in those genetic diseases that cause irreversible damage to organs *in utero*, targeting of stem cell progenitors that are abundant and accessible in the fetus as well as dose scaling where the small size of the fetus allows relatively high doses of cells to be delivered. There is an opportunity for engraftment of donor cells without the need for myeloablation due to the immature status of the fetal immune system prior to thymic processing of self-antigen, a normal event in hematopoietic ontogeny (Waddington et al., 2007; David and Peebles, 2008; Shaw et al., 2011). Furthermore, immunological tolerance would allow postnatal reinfusion of cells to boost the effect after birth, as demonstrated recently in two children with osteogenesis imperfecta treated by IUT using MSCs (Götherström et al., 2014). Postnatal reinfusion with more MSCs from the original infusion source resulted in improved growth rate after birth.

As with any new therapeutic modality, the risks of IUT are not well characterized and the efficacy is still to be determined for some diseases. For *in utero* gene therapy, where vectors are given directly to the fetus for correction of genetic disease there has been direct guidance given by the NIH Recombinant DNA advisory committee report (RAC, 2000) on a pre-proposal for the initial application. The recommendations included that treatment should be limited only to: diseases that carry serious morbidity and mortality risks for the fetus either *in utero* or postnatally, do not have an effective postnatal therapy, or have a poor outcome using available postnatal therapies, can be definitively diagnosed *in utero* and have a well-defined genotype/phenotype relationship, have an animal model for *in utero* gene transfer that recapitulates the human disease and that the therapy would correct all serious abnormalities. It was recognized that a direct fetal vector injection approach would be difficult to justify given the above.

A combination IUSCGT approach however seems more likely to be acceptable. The UK Gene Therapy Advisory Committee (GTAC) considered this in their broader judgments about gene therapy *in utero* (Eckstein, 2003). The New and Emerging Technologies subgroup of GTAC found that the use of genetically modified stem cells in stem cell transplantation to the fetus was a possibility stating “such *ex vivo* modification would be unlikely to carry with it any higher risk to the germ line than the trials of postnatal somatic gene therapy which have already been approved.”

### DEVELOPMENT OF THE FETAL IMMUNE SYSTEM AND BARRIERS TO ENGRAFTMENT AFTER IUT

The fetal immune system is commonly regarded as immature and unresponsive despite reports showing its functional immune response (Mold and McCune, 2012). IUT relies crucially on the concept that the developing fetal immune system might accept a foreign cell or antigen and become tolerant to it. The presence of human NK cells have been detected as early as gestational week 6 in the fetal liver and in the fetal spleen at gestational week 15 (Phillips et al., 1992). Fetal NK cells have the ability to differentiate early *in utero* and are highly responsive to cytokines and antibody-mediated stimulation, and have shown to be functionally immature compared to adult NK cells (Phillips et al., 1992; Ivarsson et al., 2013).

The immune system during early gestation undergoes a process of self-education that occurs in the thymus. The positive and negative selection of pre-lymphocytes for the recognition of “self” major histocompatibility complex (MHC) antigen allows a repertoire of lymphocytes to be capable of direct and indirect antigen presentation which results in the deletion of alloreactive T-cells, regulatory T cells (Tregs) enrichment and creates donor-specific immune tolerance (Nijagal et al., 2013). Thus, to prevent limited engraftment, transplants should be introduced prior to the appearance of mature T-cells in the fetal thymus (Peranteau et al., 2006; Roybal et al., 2010; Nijagal et al., 2013).

In the human fetus, the immune system develops from 12 to 14 weeks of gestation, when profound increases in circulating T lymphocytes can be observed (Darrasse-Jèze et al., 2005; Takahama, 2006). Delivery of gene therapy may be required before this gestational age, which currently could limit the routes of application that can be safely used, although advances in engineering and imaging is leading to large improvements in fetal imaging and injection systems. It was demonstrated that the human fetus may have developed a functional immune system during the second trimester of gestation (Tse et al., 2005). Hematological compositions of human fetal blood and liver between 8 and 17 weeks gestation showed an increase in fetal red blood cell, white blood cell, and platelet counts with advancing gestation reflecting hematologic development (Pahal et al., 2000). An increase in circulating and hepatic T lymphocytes showed the presence of thymic maturation before the 13th week of gestation while the proportion of circulating primitive hematopoietic stem and progenitor cells decreased after each successive gestational week. These findings support the concept of introducing IUT before the 13th week of gestation to induce actively acquired specific tolerance to the foreign antigen (Pahal et al., 2000). Thus, IUT could be performed to the corresponding hematopoietic compartments or systemically depending on the gestational age the transplantation occurs (Tavian and Peault, 2005).

Studies in mice strongly support there being an immune barrier to allogeneic engraftment after IUHCT. Transplantation of allogenic HSCs at day 14 post conception gave initially similar results to IUT with congenic HSCs at 1 week of age (100%) but after 6 months, engraftment dropped rapidly (19% allogenic versus 100% congenic; Muench, 2005; Tse et al., 2005; Peranteau et al., 2007; Shaw and Bollini, 2011). Strategies to improve engraftment of allogenic HSC *in utero* have include the use of busulfan, cotransplantation of LLME-treated, MHC-sensitized donor lymphocytes, CD26 inhibition and using haploidentical HSC sources (Hayashi et al., 2004; Ashizuka et al., 2006; Vrecenak et al., 2014). For instance, low-levels of allogeneic chimerism could be enhanced to near-complete donor chimerism in murine models by postnatal minimally myeloablative total body irradiation (TBI) followed by same-donor BM transplantation (Peranteau et al., 2002). Due to the concerns with toxicity, minimally toxic postnatal regimens such as busulfan conditioning have been studied and shown to improve therapeutic levels of allogeneic engraftment (Ashizuka et al., 2006). Mice with <1 and >1% chimerism, had 60 and 100% enhanced engraftment, respectively (Ashizuka et al., 2006).

Maternal T cells play a key role in the success *in utero* therapy by being a barrier to engraftment (Nijagal et al., 2011). There were no differences observed in engraftment of syngeneic and allogeneic fetal recipients when cells were matched to the mother in a murine model. It is believed that the immune barrier may result from maternal pathogenic immune responses as a result of pro-inflammatory signals released during fetal intervention (Nijagal et al., 2011). Recent studies of canine IUT with HSCs *in utero* were encouraging. A time of 40 days gestation (term 63 days) was chosen for these experiments since it was at the initiation of thymic selection, and prior to BM hematopoiesis, therefore being optimal for engraftment. Intracardiac injection was the most efficient delivery method giving much higher levels of donor cell engraftment than intraperitoneal injection. The authors achieved stable long-term multilineage engraftment in 21 of 24 surviving recipients with an average level of initial chimerism of 11.7% (range 3–39%) without conditioning and with no evidence of graft versus host disease (GVHD). Donor cell chimerism remained stable for up to 2 years and was associated with donor specific tolerance for renal transplantation (Vrecenak et al., 2014). Intracardiac injection early in gestation currently carries an increased risk of miscarriage in clinical practice compared to intraperitoneal injection, but these findings suggest that clinically relevant levels of engraftment might be achievable using this approach and research is underway to evaluate safety and feasibility in relevant pre-clinical animal models prior to the first human studies. Using stem cells that are matched to the fetus, i.e., autologous cells, is an alternative approach which is discussed further on in this review.

### SEVERE COMBINED IMMUNODEFICIENCY

Severe combined immunodeficiency (SCID) has been successfully corrected by ultrasound guided intraperitoneal or intravenous fetal injection of HSCs derived from the paternal BM or an allogeneic fetal liver (Flake et al., 1996; Westgren et al., 2002). X-linked SCID is an immunodeficiency caused by the mutation of *IL2RG,* which encodes the cytokine-receptor γ chain that results in a block in T-cell development and a severe deficiency of mature T cells (Flake et al., 1996). After IUSCT, stable split chimerism with the T-cell lineage of donor origin and all other lineages of host origin was seen postnatally in treated individuals as evidence of immune system reconstitution (Flake, 2004). For most patients, the diagnosis of SCID is only made in the neonatal period meaning that postnatal treatment is the only option. Rapid advances in fetal medicine, such as the availability of non-invasive prenatal diagnosis in the first trimester is likely to make prenatal screening a reality. IUSCT currently is an option for affected families that have a one in four risk of recurrence, where first trimester prenatal diagnosis can be made by CVS leaving time to perform stem cell transplantation using allogeneic stem cells. The most common treatment for SCID patients is a postnatal BM transplant where a matched donor is required. More recently, where a suitable donor is not available, a stem cell gene therapy approach has used gene corrected autologous BM transplantation with great success (Demaison et al., 2002; Gaspar and Thrasher, 2005; Thrasher et al., 2006; Gaspar et al., 2011; Montiel-Equihua et al., 2012). For instance, in 2012, around 30 patients had been treated most of whom had experienced clinical benefit with the absence of any vector-related complications (Gaspar and Thrasher, 2005). There is a chance of insertional mutagenesis occurring during retroviral and lentiviral vector integration into host-cell chromosomes as well as the development of lymphoproliferative disease in individuals with SCID (Gaspar and Thrasher, 2005; Yáñez-Muñoz et al., 2006; Howe et al., 2008). It is important for clinical therapies to achieve stable transgene expression while minimizing insertional mutagenesis (Baum et al., 2003). Integration-deficient lentiviral vectors and self-inactivating (SIN) gammaretroviral vectors have a low risk and in cellular and *in vivo* models of SCID can mediate stable transduction (Yáñez-Muñoz et al., 2006; Thornhill et al., 2008).

### CONGENITAL BLOOD DISORDERS

Inherited blood disorders such as the hemoglobinopathies or clotting disorders would be a relatively simple target for IUT as the fetal circulation can be reached through the umbilical vein (UV) at the placental cord insertion or the intrahepatic UV, or even via the peritoneal cavity, a route used successfully to transfuse anemic fetuses.

Prenatal screening and diagnostic services for congenital hemoglobinopathies are available in many countries making them an attractive option for an *in utero* therapeutic approach (David and Waddington, 2012). Prenatal diagnosis can be achieved currently from 11 weeks of gestation using CVS, or amniocentesis from 15 weeks, but increasingly there are advances in non-invasive prenatal screening and diagnosis using circulating fetal DNA detected in the maternal plasma allows the diagnosis of congenital disorders as early as 7 weeks (Lo et al., 1998). Since AF or chorionic villus samples are accessible relatively easily and early in pregnancy, they would provide the potential for therapeutic use after clinical prenatal diagnosis have been performed.

Inherited abnormalities of hemoglobin (Hb), a tetramer of two α-like and two β-like globin chains, are a common and global problem. Over 330,000 affected infants are born annually worldwide, 83% with sickle cell disorders and 17% with thalassemias (Modell and Darlison, 2008). Current treatment of β-thalassemia is by postnatal allogeneic hematopoietic stem cell transplantation (HSCT) which can cure the condition with recent results of 90% survival and 80% thalassemia-free survival (Angelucci et al., 2000). However, this option is only available in approximately 30% of cases due to the lack of a suitable matched donor (Lucarelli, 2002), and it is associated with complications such as GVHD. For children where HSCT is unavailable, they are dependent on blood transfusions that result in iron overload, and the need for iron chelation therapy. In alpha-thalassemia, some individuals who make very little or no α globin chains, have severe anemia, termed Hb Bart’s hydrops fetalis syndrome which is commonly diagnosed prenatally and if untreated causes death in the neonatal period (Harteveld and Higgs, 2010). Current treatment of sickle cell relies on a number of strategies such as the use of prophylactic antibiotics, pneumococcal vaccination and good hydration, and effective crisis management such as using oxygen and pain-relief (Meremikwu and Okomo, 2011).

Attempts to cure thalassemia and sickle cell disease using gene therapy have been hampered by the large globin gene and globin promoters that are difficult to accommodate within vector systems. Amelioration or even cure of mouse models of human sickle cell disease (Pawliuk et al., 2001) and β-thalassemia major (Pawliuk et al., 2001; Persons et al., 2003; Puthenveetil et al., 2004) has been achieved using lentivirus vectors that contain complex regulatory sequences from the LCR region. Recent advances in vector design have improved gene transfer for the hemoglobinopathies such as the ubiquitous chromatin opening element (UCOE) augmented spleen focus forming virus (SFFV) promoter/enhancer which provides lentivirus vectors with a natural tropism for the hematopoietic system (Antoniou et al., 2003; Williams et al., 2005) resulting in reproducible and stable function in BM and all differentiated peripheral hematopoietic cell lineages (Zhang et al., 2007).

Clotting disorders are caused by deficiencies in coagulation factors, for example, hemophilia B, which is due to mutations in the factor IX (F9) gene resulting in a deficiency in the blood clotting protein human factor IX (hFIX; Waddington et al., 2004b). The current treatment offered to patients with inherited coagulopathies includes lifelong recombinant protein infusions, which is required to avoid major pathology, and it is an expensive and limited resource. In some patients, protein infusions can also lead to the formation of antibodies to the infused product, which prevents its use. Gene therapy cure of inherited coagulopathies has come closer to reality with the use of adeno-associated virus vectors (AAV). Animal experiments have shown AAV to be a promising vector system and this has led to the first human trials for this disease by applying AAV-hFIX intramuscularly to eight adult patients with severe hemophilia B which showed a small increase in hFIX plasma levels and a reduction in exogenous protein requirement (Kay et al., 2000; Manno et al., 2003). More recently, one trial used a self-complimentary AAV-hFIX vector that gives higher levels of transgenic protein expression *in vivo* than earlier single-stranded vectors. A single peripheral vein infusion of a serotype-8-pseudotyped, self-complementary AAV vector expressing a codon-optimized hFIX transgene in six patients with severe hemophilia B (FIX activity, <1% of normal values) gave FIX expression at 2–11% of normal levels in all participants. A short course of glucocorticoid therapy normalized raised liver enzyme levels that were observed in two patients (Nathwani et al., 2011). AAV vectors with hFVIII and hFVII are becoming available and are being tested in pre-clinical studies (Binny et al., 2012; McIntosh et al., 2013).

Proof of principle studies have shown long-term expression of hFIX proteins at therapeutic levels and induction of immune tolerance (Waddington et al., 2007) after *in utero* gene therapy using lentiviral vectors in mice (Waddington et al., 2004a,b). More recently, using the same self-complementary AAV8 vector expressing the human factor IX (hFIX) gene used for the clinical trials, long-term hFIX expression was observed after ultrasound guided intraperitoneal injection of fetal sheep in early and late gestation (Nathwani et al., 2006; David et al., 2011). No functional antibodies could be detected against the vector or transgene product and no liver toxicity was observed. Antibodies to the therapeutic gene were detectable when the animals were challenged at 6 months of age postnatally with the hFIX recombinant protein, showing that induction of immune tolerance was not achieved. This was probably due to the fall in hFIX expression that was undetectable by 1 year after birth. UV delivery in fetal non-human primates of a 10-fold higher dose of the same self-complementary AAV system in late gestation produced clinically relevant levels of hFIX sustained for over a year, with liver-specific expression and a non-neutralizing immune response (Mattar et al., 2011).

#### In utero transplantation for congenital blood disorders

In comparison to the relative success of postnatal transplantation for blood disorders, results of clinical cases of IUT to cure blood disorders have been disappointing (Nijagal et al., 2012; Pearson and Flake, 2013). For instance, the transplantation of CD34^+^ cells from either fetal liver or adult BM in cases of hemoglobinopathies, showed no evidence of engraftment in all 22 cases with a clinical outcome of lifelong blood transfusion or disease-related mortality (Tiblad and Westgren, 2008). Attempts to treat other diseases such as sickle cell or metabolic storage disorders have been unsuccessful after fetal liver-derived stem cell transplantation, even wherein a suitably matched donor has been available (D’Azzo, 2003; Westgren, 2006).

Transplantation of autologous derived fetal liver stem cells has been attempted in the fetal sheep. Fetal liver stem cells collected from first trimester preimmune sheep fetuses using ultrasound-guided hepatic sampling were labeled with PKH26 and then transplanted intraperitoneally into allogeneic and autologous fetal recipients. Engraftment of donor cells was equivalent after autologous or allogeneic transplantation (up to 4.7% in fetal liver, spleen, BM, blood, and thymus) but the fetal loss rate was high (29% allogeneic and 73% autologous transplantation) making this technique difficult to justify in clinical practice (Schoeberlein et al., 2004).

For coagulopathies, transplantation of MSCs may be a feasible therapeutic option. In a sheep model of hemophilia A that recapitulates the human condition (spontaneous bleeds and debilitating hemarthroses), encouraging results were found after postnatal intraperitoneal infusion of paternally derived MSCs transduced with a porcine FVIII-encoding lentiviral vector. Infusions of factor VIII were no longer required and damaged joints were fully recovered. However, a sharp increase in pre-existent antibodies occurred with time following transplantation which decreased the effectiveness and limited the duration of therapy (Porada et al., 2011). This emphasizes the need for an IUSCT approach for this condition.

#### In utero stem cell gene therapy for congenital blood disorders using AF-derived stem cells

**FIGURE 1:**
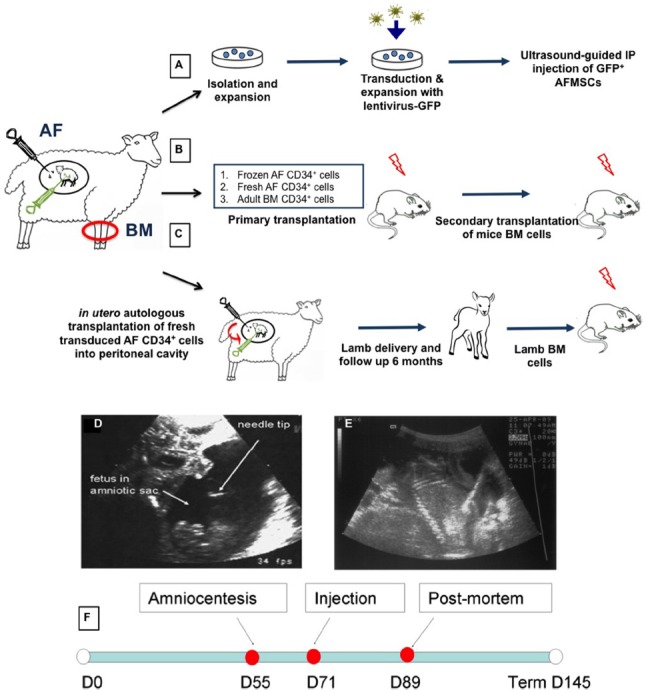
**Experimental design and injection procedure.** After amniocentesis collection, amniotic fluid mesenchymal stem cells (AFMSCs) were cultured in adherence in defined conditions. Cells were transfected with lentivirus GFP and re-injected into the peritoneal cavity of the fetal donor **(A)**. Transduced sheep eGFP^+^ CD34^+^ selected from fresh or frozen AF and adult BM cells were transplanted into immunocompromised NSG mice (primary and secondary xenogeneic transplantation) **(B)**. Transduced sheep eGFP^+^CD34^+^ fresh AF were also injected into donor sheep fetuses (*in utero* autologous transplantation) that were subsequently delivered and followed for up to 3 months of age. Bone marrow from these primary sheep recipients was then used to perform xenogeneic secondary transplantation into NSG mice **(C)**. AF, amniotic fluid; BM, bone marrow. Sonograms showing ultrasound guided amniocentesis **(D)** and intraperitoneal injection **(E)**. Amniocentesis was performed using a 22 gauge needle to collect 10 ml amniotic fluid from the amniotic cavity around a fetal sheep at 58 days of gestation. For intraperitoneal injection of transduced expanded amniotic fluid cells we used a 20 gauge needle inserted through the anterior abdominal wall of a sheep fetus at 76 days of gestation. Echogenicity can be seen throughout the peritoneal cavity after injection of cells. Scale bars: 5 cm. **(F)** Timeline of the experiment.

**FIGURE 2:**
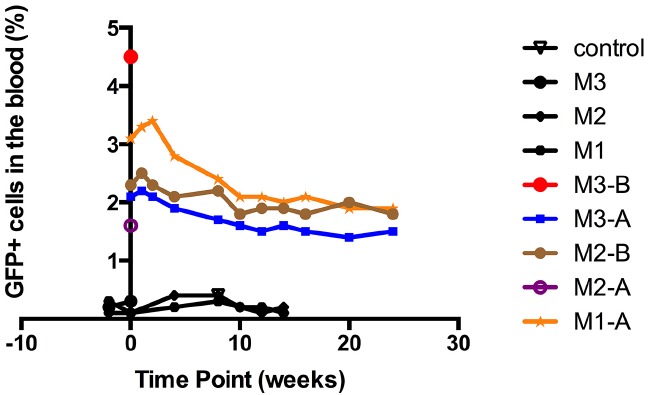
***In utero* autologous intraperitoneal transplantation of sheep amniotic fluid CD34^+^ cells in fetal sheep with long-term follow up.** Engraftment in the peripheral blood after *in utero* transplantation of autologous sheep CD34^+^eGFP^+^ AF cells. All five born lambs showed eGFP^+^ cells in the peripheral blood at birth (M1-A, M2-A, M3-A, M2-B, and M3-B), and all three survivors revealed persistent levels of engraftment of around 2% that persisted up to the last sampling point at 6 months of age (M1-A, M2-B, and M3-A). Negative control: peripheral blood from uninjected sheep. M1, M2, and M3: the three ewes that showed negativity for eGFP signal.

**FIGURE 3:**
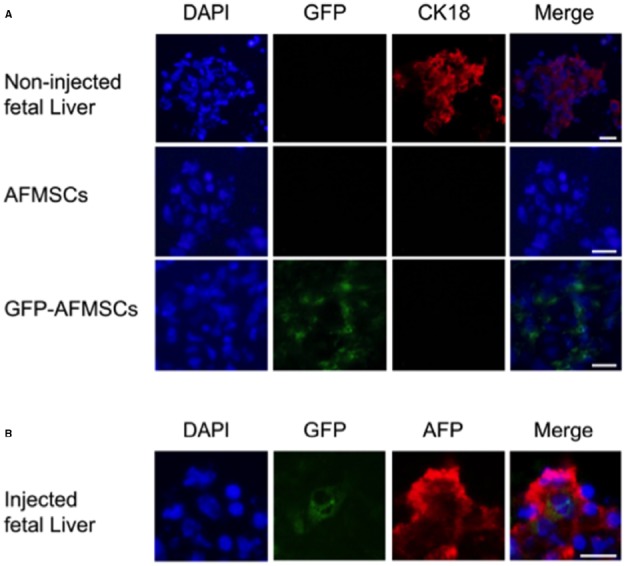
**Immunofluorescence for CK18 and AFP expression in fetal liver and amniotic fluid stem cells.** Panel **(A)** shows positive CK18 expression in cells cultured from a control fetal sheep liver but no expression in amniotic fluid mesenchymal stem cells (AFMSCs) or transduced cells (GFP-AFMSCs) before injection. An uninjected sheep fetus of comparable gestational age was used as the control. Panel **(B)** shows co-expression of GFP with expression of Alpha fetoprotein (AFP) another liver specific marker in the fetal liver after transplantation of transduced AFMSCs. Scale bars: 20 *µ*m.

Given the concerns around *in utero* application of gene therapy directly to the fetus, our group have been studying whether a combination of autologous transplantation with gene corrected AFS might be a potential therapeutic approach. We have studied the functional hematopoietic potential of transduced green fluorescent protein (GFP)^+^ sheep AF-derived stem cells, before and after autologous IUSCT. First trimester sheep AF was collected by ultrasound-guided amniocentesis or at post mortem examination. We used a novel sheep CD34^+^ antibody that allows flow cytometric detection of sheep HSC/progenitors present within BM, cord blood, and mobilized peripheral blood. This antibody also enriches for HSC/progenitors with enhanced *in vitro* colony-forming potential (Porada et al., 2008). Sheep CD34^+^ AF or adult BM cells were selected and transduced overnight with an HIV lentivirus vector containing eGFP. Transduced fresh or frozen CD34^+^ AF, or BM cells, were injected intravenously into NOD-SCID-gamma (NSG) mice. GFP^+^ cells were detected in the hematopoietic organs and peripheral blood of NSG mice primary and secondary recipients 3 months later (Figure 1). Autologous IUSCT was performed in fetal sheep using ultrasound-guided intraperitoneal injection of fresh transduced GFP^+^ CD34^+^AF cells. GFP^+^ cells were detected in the peripheral blood of injected lambs up to 6 months postnatally (Figure 2) and 3 months after secondary transplantation of BM from autologous IUSCT lambs into NSG mice, GFP^+^ cells were detected in hematopoietic organs. This demonstration of autologous IUSCT of CD34^+^AF cells in a large animal model supports the concept for clinical translation to treat congenital hematopoietic diseases *in utero* (Shaw et al., 2014).

Human β-thalassemia iPS have now been generated from AFS using a single excisable lentiviral stem cell cassette vector. AFS from the prenatal diagnosis of a β-thalassemia patient were reprogrammed by expression of the four human reprogramming factors Oct4, KLF4, SOX2, and c-MYC using a doxycycline lentiviral system and demonstrated teratoma formation (Fan et al., 2012). There are concerns that these iPS cells may be more likely to develop teratomas than AFS cells that have a low risk of this complication. This type of cell manipulation however, may provide clinicians with corrected autologous patient-specific iPS cells to use in a combination IUSCGT approach for the treatment of thalassemia (Fan et al., 2012).

### *IN UTERO* TRANSPLANTATION FOR OTHER CONGENITAL DISORDERS

IUT is a possible treatment strategy for congenital disorders that affect organ systems other than the blood. These include myelomeningocele (MMC; Danzer et al., 2012) which represents the most severe form of spina bifida, cystic fibrosis (CF), lysosomal storage diseases such as acute neuronopathic (type II) Gaucher disease, neuronal ceroid lipofuscinoses, and Niemann–Pick disease type C, ornithine transcarbamylase deficiency (OTC), as well as muscular dystrophy (David et al., 2003). Many of these diseases and organ systems would be amenable to IUT using MSCs, which compared to HSCs, are less immunologically competent and may result in less transplantation related rejection (O’Donoghue and Fisk, 2004).

#### In utero transplantation of mesenchymal stem cells

Human BM-MSCs have shown to have long-term engraftment and have the ability to differentiate into various tissues when transplanted into fetal sheep (Mackenzie and Flake, 2001). The therapeutic potential for combining surgical repair and transplantation of MSCs *in utero* has been demonstrated recently for the treatment of spina bifida in a rat model (Li et al., 2012). IUT of first trimester human fetal blood MSC ameliorates the skeletal disorder in a mouse model of osteogenesis imperfecta (Guillot et al., 2008). IUT of fetal MSCs reduced fracture rates and skeletal abnormalities (Guillot et al., 2008). Two cases of IUT using allogenic fetal liver MSC in the third trimester had encouraging results with a successful engraftment which demonstrated 7.4% chimerism at 9 months of age in one case and good long-term outcomes (Le Blanc et al., 2005; Götherström et al., 2014).

#### In utero stem cell gene therapy using amniotic fluid-derived MSCs

High fetal survival was found after intraperitoneal injection of autologous AFMSCs in the sheep (Mehta et al., 2011; Shaw and Bollini, 2011). AF was collected under ultrasound-guided amniocentesis in early gestation pregnant sheep (*n* = 9, 58 days of gestation, term = 145 days) and AFMSCs were isolated, expanded, and transduced using an HIV vector encoding enhanced GFP with 63.2% (range 38.3–96.2%) transduction efficiency rate (Figure 1). Transduced AFMSCs were injected into the peritoneal cavity of each donor fetal sheep at 76 days under ultrasound guidance with a 78% overall survival rate for the full procedure. After 2 weeks, GFP^+^ cells and protein was detected in fetal tissues including liver, heart, placenta, membrane, umbilical cord, adrenal gland, and muscle and this was further confirmed by cytofluorimetric and immunofluorescence analysis (Figure 3).

AFS cells intraperitoneally injected in a necrotizing enterocolitis rat model had shown improved survival, clinical status, gut structure, and function (Eaton et al., 2013; Zani et al., 2014). These findings suggest that the use of AFS cells as a source of cells for *in utero* therapy could be an alternative way of ameliorating prenatal congenital disease.

## ROUTE TO THE CLINIC

Preclinical testing in animal models of disease will be an important step before clinical translation is realized. There is no ideal animal model and a balance is needed, taking into consideration the gestational development of the organ to be targeted and how that relates to its development in the human, the type of placentation, fetal size, number and lifespan, parturition, and the fetal and maternal immune response (Mehta et al., 2012; Mehta and Abi-Nader, 2012). An assessment of the safety, accessibility, transduction efficiency, and behavior of various stem cells (i.e., cord blood, placenta, AF, fetal tissue) *in vitro* as well as in the fetal environment are required to evaluate proof-of-principle strategies based on gene transfer or cell transplantation into the fetus to ensure accurate organ-directed manipulation and delivery (Moreno et al., 2012). Thus, the efficacy of treatment can be evaluated from murine models to large animal models such as sheep and primates (Mehta et al., 2012; Mehta and Abi-Nader, 2012).

Toxicology studies will be needed using animals such as the pregnant rabbit, in which reproductive toxicology is commonly performed, with good historical datasets and a model that is understood by the regulators. A variety of guidelines and regulations such as those described by the Committee for Medicinal Products for Human Use (CHMP) of the European Medicines Agency will need to be taken into consideration when planning preclinical study protocols. These could include for example, the guidelines on the non-clinical testing for inadvertent germline transmission of gene transfer vectors (EU, 2006) or on the non-clinical studies required before first clinical use of gene therapy medicinal products (European Medicines Agency, 2007).

In addition to animal studies, the safety of gene therapy vectors has to be evaluated. Integration site analysis has become a critical tool to measure the “vector-on-host” and “host-on-vector” effects in gene therapy (Biasco et al., 2011). Also, models such as the human placenta can be utilized *in vitro* as it would provide a wealth of data on the physiology of normal and pathological human placentae and may be useful in measuring the spread of vector from the fetus to the mother or vice versa.

## CONCLUSION

Advances in prenatal screening and molecular diagnosis have provided the ability of detecting the majority of genetic diseases early in gestation. Early diagnosis provides the option of possible treatment options that can be explored either at the prenatal or postnatal period depending on the condition. The option of preemptive treatment of congenital diseases *in utero* by stem cell or gene therapy are encouraging as it changes the perspective of congenital diseases. However, further work focusing on the safety and ethical issues need to be addressed before clinical applications can be considered.

## AUTHOR CONTRIBUTIONS

Durrgah L. Ramachandra, Steven S. W. Shaw, Panicos Shangaris, Stavros Loukogeorgakis, Pascale V. Guillot, Paolo De Coppi, and Anna L. David made substantial contributions to the conception and design of the work. Durrgah L. Ramachandra and Panicos Shangaris drafted the work and Paolo De Coppi and Anna L. David revised it critically for important intellectual content. Durrgah L. Ramachandra, Steven S. W. Shaw, Panicos Shangaris, Stavros Loukogeorgakis, Pascale V. Guillot, Paolo De Coppi, and Anna L. David gave final approval of the version to be published and agreed to be accountable for all aspects of the work in ensuring that questions related to the accuracy or integrity of any part of the work are appropriately investigated and resolved.

### Conflict of interest statement

Anna L. David is an unpaid consultant and director of Magnus Growth, part of Magnus Life Science, which is aiming to take to market a novel treatment for fetal growth restriction.
