# Corrigendum: *In utero* therapy for congenital disorders using amniotic fluid stem cells

**DOI:** 10.3389/fphar.2015.00039

**Published:** 2015-03-03

**Authors:** Durrgah L. Ramachandra, Sheng-Wen Steven Shaw, Panicos Shangaris, Stavros Loukogeorgakis, Pascale V. Guillot, Paolo De Coppi, Anna L. David

**Affiliations:** ^1^Stem Cells and Regenerative Medicine, Institute of Child Health, University College LondonLondon, UK; ^2^Department of Obstetrics and Gynaecology, Chang Gung Memorial Hospital at LinkouTaoyuan, Taiwan; ^3^Department of Obstetrics and Gynaecology, College of Medicine, Chang Gung UniversityTaoyuan, Taiwan; ^4^Prenatal Therapy, Institute for Women's Health, University College LondonLondon, UK; ^5^Cellular Reprogramming and Perinatal Therapy, Institute for Women's Health, University College LondonLondon, UK

**Keywords:** amniotic fluid, stem cell, transplantation, prenatal, cell therapy

I am writing this manuscript to correct the second author information. My name is correctly spelt, S.W. Steven Shaw. But “S.W. Steven” is the first name, “Shaw” is the last name. My publications are always searched by “Shaw SW” in pubmed. However, in this article, my name is searched by “Shaw SS.”

## Conflict of interest statement

The authors declare that the research was conducted in the absence of any commercial or financial relationships that could be construed as a potential conflict of interest.

